# Prehospital induction of mild hypothermia with cold normal saline for cardiac arrest: more harm than good?

**DOI:** 10.1186/s13054-014-0559-0

**Published:** 2014-10-09

**Authors:** Vishal Yajnik, Hernando Gomez

**Affiliations:** Department of Critical Care Medicine, University of Pittsburgh, Scaife Hall, 3550 Terrace Street, Pittsburgh, PA 15261 USA

## Expanded abstract

### Citation

Kim, Francis, Graham Nichol, Charles Maynard, Al Hallstrom, Peter J. Kudenchuk, Thomas Rea, Michael K. Copass et al. “Effect of prehospital induction of mild hypothermia on survival and neurological status among adults with cardiac arrest: a randomized clinical trial.” JAMA 311, no. 1 (2014): 45–52. PubMed PMID: 24240712.

### Background

Hospital cooling improves outcome after cardiac arrest, but prehospital cooling immediately after return of spontaneous circulation may result in better outcomes.

### Methods

*Objective:* To determine whether prehospital cooling improves outcomes after resuscitation from cardiac arrest in patients with ventricular fibrillation (VF) and without VF.

*Design:* A randomized clinical trial that assigned adults with prehospital cardiac arrest to standard care with or without prehospital cooling. Patient follow-up was completed by May 1, 2013. Nearly all of the patients resuscitated from VF and admitted to the hospital received hospital cooling regardless of their randomization.

*Setting: *King County, Washington.

*Subjects:* Adults with prehospital cardiac arrest and resuscitated by paramedics were eligible and 1359 patients (583 with VF and 776 without VF) were randomized between December 15, 2007, and December 7, 2012.

*Intervention:* Infusing up to 2 L of 4°C normal saline to patients with prehospital cardiac arrest as soon as possible following return of spontaneous circulation.

*Outcomes:* The primary outcomes were survival to hospital discharge and neurological status at discharge.

### Results

The intervention decreased mean core temperature by 1.20°C (95% CI, −1.33°C to −1.07°C) in patients with VF and by 1.30°C (95% CI, −1.40°C to −1.20°C) in patients without VF by hospital arrival and reduced the time to achieve a temperature of less than 34°C by about 1 hour compared with the control group. However, survival to hospital discharge was similar among the intervention and control groups among patients with VF (62.7% [95% CI, 57.0%-68.0%] vs 64.3% [95% CI, 58.6%-69.5%], respectively; P = 0.69) and among patients without VF (19.2% [95% CI, 15.6%-23.4%] vs 16.3% [95% CI, 12.9%-20.4%], respectively; P = 0.30). The intervention was also not associated with improved neurological status of full recovery or mild impairment at discharge for either patients with VF (57.5% [95% CI, 51.8%-63.1%] of cases had full recovery or mild impairment vs 61.9% [95% CI, 56.2%-67.2%] of controls; P = 0.69) or those without VF (14.4% [95% CI, 11.3%-18.2%] of cases vs 13.4% [95% CI,10.4%-17.2%] of controls; P = 0.30). Overall, the intervention group experienced rearrest in the field more than the control group (26% [95% CI, 22%-29%] vs 21% [95% CI, 18%-24%], respectively; P = 0.008), as well as increased diuretic use and pulmonary edema on first chest x-ray, which resolved within 24 hours after admission.

### Conclusions

Although use of prehospital cooling reduced core temperature by hospital arrival and reduced the time to reach a temperature of 34°C, it did not improve survival or neurological status among patients resuscitated from prehospital VF or those without VF.

## Commentary

Out of hospital cardiac arrest is a leading cause of death in the United States, is associated with high mortality and morbidity, and represents a massive cost burden to the health system. Out of multiple investigational strategies attempting to ameliorate post-cardiac arrest neurological injury, the induction of mild hypothermia has been proven in clinical trials to be one of the most effective [3,4] and has been widely adopted in international guidelines [5]. However, the available data has left several questions unanswered [6], most notably regarding its therapeutic value [7], and this study by Kim et al. sheds light on the effects of inducing pre-hospital hypothermia on all cardiac arrest.

The present study is a large, non-concealed, randomized controlled trial that aimed to investigate the impact of induction of pre-hospital hypothermia on survival and neurological outcomes in patients with and without ventricular fibrillation (VF). The authors randomized 1359 adult patients to standard of care with or without pre-hospital induction of hypothermia. The intervention arm received up to 2 liters of 4°C normal saline by paramedics as soon as return of spontaneous circulation (ROSC) was achieved with a goal temperature of 34°C. Hypothermia was induced in all such patients upon arrival to the hospital per institution protocols regardless of study allocation, with the primary endpoint being survival and neurologic status at discharge.

Infusion of cold saline shortened the time needed to achieve the targeted temperature in patients who received pre-hospital cold saline infusion. However, there was no difference in survival or neurologic outcome, regardless of presenting rhythm. In contrast to prior studies, the authors found a significant increase in the incidence of rearrest, pulmonary edema and the use of diuretics in the intervention group.

The quality and timing of bystander cardiopulmonary resuscitation, as well as the performance of the emergency medical services are critical elements of the process of caring for cardiac arrest victims, and are direct determinants of survival and functional outcomes. Accordingly, regional differences in performance that account for wide variations in survival rates across the nation may directly influence the impact any given treatment may have on outcome. The lack of survival advantage in the intervention group found by Kim et al. must be taken in the context of the region and health system where the study was developed. Kim et al. report mortality rates for VF and non-VF arrest of 35.7 and 83.7%, respectively, which are among the best in the United States. Could a system with less efficient emergency medical services than the one studied by Dr. Kim’s group in this study potentially benefit from such intervention? If longer transit times were needed for patients to arrive at hospital centers that could provide targeted temperature control, would achieving the colder temperature in an expedited manner as seen in the intervention group in this study prove beneficial for survival or neurological outcome? Scales et al. are presently conducting a study in Ontario, Canada with pre-hospital hypothermia in cardiac arrest patients in a broader area, with longer transit times, which may provide some answers to these questions [10].

Alternatively, any beneficial effect derived from hypothermia in the study by Kim et al. may have been obscured by the intervention group having higher rates of rearrest, which would undoubtedly induce secondary neurologic injury and negatively impact outcome. Yannopolous et al. found that in swine, the induction of hypothermia using intravenous volume loading has been associated with significantly decreased coronary artery perfusion pressure compared with surface cooling methods. Interestingly, they also found that intra-cardiopulmonary resuscitation cooling resulted in a decreased myocardial infarct size [11]. Perhaps, intra-cardiopulmonary resuscitation cooling, rather than post-arrest cooling, could be beneficial for this patient population, and it is possible that the increased rate of re-arrest may have been partly due to volume loading.

The study has multiple strengths; here we have a large scale, randomized controlled trial, with a low cost, and potentially generalizable intervention for a lethal clinical and public health problem. The authors completed their planned follow-up and established a priori specific subgroup analysis. In addition, the impact of randomization on in-hospital clinical decisions was assessed by performing post-hoc analyses of the rates of withdrawal of life support, use of angiography or a change in the level of life support between groups and demonstrating with it a similar degree of between groups. Limitations include, as with most studies examining therapeutic hypothermia, lack of provider blinding to the intervention. These findings may be applicable in places with similar EMS delivery quality as King County, but it is still unclear that these results are generalizable to other regions with lesser capabilities.

## Recommendation

The results of this study suggest that in the setting of a well organized, highly efficient emergency medical system, induction of pre-hospital hypothermia using 4°C normal saline does not improve survival or neurologic outcome, and may be associated with an increased rate of rearrest, pulmonary edema and use of furosemide in the first 6 hours after admission to the hospital. Whether these findings are generalizable to other environments is still unknown, as it is unclear whether these findings are the result of the method of induction rather than the physiologic principle of the intervention. Certainly, more research in this area is needed before hypothermia is excluded as a beneficial pre-hospital intervention, and we look forward to future developments in this area.
